# Cross-sectional evaluation of a longitudinal consultation skills course at a new UK medical school

**DOI:** 10.1186/1472-6920-11-55

**Published:** 2011-08-08

**Authors:** Alexia Papageorgiou, Susan Miles, Michelle Fromage, Julie Kemmy, Sam J Leinster

**Affiliations:** 1 Norwich Medical School, Faculty of Medicine and Health Sciences, University of East Anglia, Norwich Research Park, Norwich, NR4 7TJ, UK

## Abstract

**Background:**

Good communication is a crucial element of good clinical care, and it is important to provide appropriate consultation skills teaching in undergraduate medical training to ensure that doctors have the necessary skills to communicate effectively with patients and other key stakeholders. This article aims to provide research evidence of the acceptability of a longitudinal consultation skills strand in an undergraduate medical course, as assessed by a cross-sectional evaluation of students' perceptions of their teaching and learning experiences.

**Methods:**

A structured questionnaire was used to collect student views. The questionnaire comprised two parts: 16 closed questions to evaluate content and process of teaching and 5 open-ended questions. Questionnaires were completed at the end of each consultation skills session across all year groups during the 2006-7 academic year (5 sessions in Year 1, 3 in Year 2, 3 in Year 3, 10 in Year 4 and 10 in Year 5). 2519 questionnaires were returned in total.

**Results:**

Students rated Tutor Facilitation most favourably, followed by Teaching, then Practice & Feedback, with suitability of the Rooms being most poorly rated. All years listed the following as important aspects they had learnt during the session:

• how to structure the consultation

• importance of patient-centredness

• aspects of professionalism (including recognising own limits, being prepared, generally acting professionally).

All years also noted that the sessions had increased their confidence, particularly through practice.

**Conclusions:**

Our results suggest that a longitudinal and integrated approach to teaching consultation skills using a well structured model such as Calgary-Cambridge, facilitates and consolidates learning of desired process skills, increases student confidence, encourages integration of process and content, and reinforces appreciation of patient-centredness and professionalism.

## Background

Good communication is an essential component of good clinical care [1]. Poor communication is a significant factor in a high percentage of complaints from patients [2]. It is widely assumed that teaching consultation skills to medical students will result in improvement in their performance in the clinical setting with a resultant reduction in the number of complaints [2]. The importance of consultation skills teaching in undergraduate medical training in terms of achieving essential outcomes for both patients and doctors has been well documented in the literature over the last ten years [2,3]. It improves medical students' communication skills in relationship building, organisation and time management, patient assessment, negotiation and shared decision-making [4]. In addition, it increases patient and clinician satisfaction, patient perceptions of control over health, preferences for an active role in health care, recall of information, adherence to recommendations, attendance and clinical outcomes [2]. In this article we present the findings of a cross-sectional evaluation study which aimed to evaluate medical students' perceptions of the consultation skills teaching sessions on a five year undergraduate MB/BS (Degree of Bachelor of Medicine and Bachelor of Surgery) programme at a new medical school.

Studies undertaken between 1970-1980 suggested that when students are taught consultation skills during the pre-clinical years of the medical degree, their confidence in their ability to communicate with patients and some consultation skills deteriorated as they progressed through medical school [5,6]. Research evidence suggests that undergraduate medical students who have been through a longitudinal communication skills curriculum achieve higher consultation skills scores on multiple station OSCEs (Objective Structured Clinical Examinations) with simulated patients than those experiencing a concentrated curriculum [7,8]. van Dalen *et al*'s [7] research suggests that "the preferred approach to communication skills training would be an integrated, longitudinal programme, which continues during the clinical years." (p. 29) This evidence is also supported by more recent research by Hook & Pfeiffer [9], who found that a more integrated communication skills curriculum with earlier patient contact "led to an earlier acquisition of communication skills and less reduction in them." (p. 155).

Faculty training in consultation skills teaching and their attitudes towards such curricula has also been investigated and shown to be very important in student learning and maintenance of such skills [8-11].

The new medical school at the University of East Anglia (UEA) received its first cohort of undergraduate medical students in 2002-3. The medical school adopted a problem based learning (PBL) curriculum. From the outset, we believed that a longitudinal approach to learning and assessing consultation skills would be more beneficial to our students than a concentrated one (when consultation skills are delivered in blocks during pre-clinical years). The course is made up of 14 system-based modules over the five years; students study 2 or 3 modules each year. In each module students generally have approximately 8 to 10 weeks of PBL-related teaching and 4 weeks in secondary care. During PBL weeks, teaching and learning are centred on detailed scenarios describing patient presentations which all relate to the module under study. For each scenario there are stated learning outcomes which all students have to achieve by the end of their PBL week. During the week they will attend small group PBL sessions to discuss the scenario, develop learning objectives and discuss their learning. They will also attend seminars covering learning specific to that week's scenario, and spend one day a week in primary care where learning opportunities at the practice will again focus on content related to that week's scenario. For example, during the Respiratory Medicine module in Year 2, the scenario for one week will focus on cough in a smoker; there will be seminars on the pathology of smoking and lung cancer, and management of lung cancer, and the learning opportunities in Primary care might focus on observing a health professional at a smoking cessation clinic or talking to a patient who has been diagnosed with lung cancer. Each module will also include a block of several weeks in secondary care where learning experiences will focus on the module as a whole, for example attending lung cancer multidisplinary team meetings and consultant-led ward rounds for emergency respiratory admissions. A unique feature of the course is early patient contact in primary and secondary care from year one which helps our students to link theory with practice. Consultation skills is one of several vertical strands that runs throughout the five years of the curriculum. Teaching on such strands takes place during the PBL week, but is not necessarily directly related to the rest of the teaching on the module. Integration is carried out as far as possible with, for example, the case scenarios used in consultation skills sessions covering the same conditions that the students are covering in that PBL week. Each strand has its own learning methods and learning outcomes.

In accordance with UK General Medical Council (GMC) and the standards set in Tomorrow's Doctors [12], the aim of our consultation skills course is to produce doctors who are well prepared to carry out patient-centred, efficient and effective medical consultations. We have adopted the Calgary-Cambridge [2] model for teaching consultation skills to undergraduate medical students; further details are provided below. To evaluate the effectiveness of the consultation skills teaching as perceived by the recipients of that teaching, the students, we have gathered and analysed systematic, anonymous feedback on all consultation skills teaching sessions since the medical degree (MB/BS) commenced in September 2002.

This article presents the results of the course evaluation questionnaire for the 2006-7 academic year. This was the first academic year where all five years of students were present. The content analysis of the open-ended question "What do you feel are the most important things you learnt during this session?" is also presented. As detailed below, one of the aims of the consultation skills course is to teach the skills under the Calgary-Cambridge model as and when they are applied to different clinical scenarios. Looking at students' responses to this question would give us an idea whether this aim is being achieved.

## Method

### The consultation skills course

As previously stated, the UEA consultation skills teaching utilises the Calgary-Cambridge model, and includes teaching on all five years of the medical degree. The Calgary-Cambridge model outlines five tasks the doctor has to achieve during the consultation (introduction, information gathering, physical examination, explanation and planning, closing the session) and two processes that take place throughout the interview (building and maintaining a therapeutic relationship and structuring the consultation). By the end of year three our students are expected to have learnt and practiced all the skills under the Calgary-Cambridge model. In years four and five these skills are revisited by using more complex scenarios (Table 1). We follow the spiral model of learning and teaching. The spiral model allows us to teach specific skills, such as those of information gathering, which the students practice during role play with simulated patients and revisit in following sessions and subsequent years [2]. The teaching is divided into two components: didactic teaching, which is carried out in lectures, and experiential sessions. During the experiential sessions small groups of 10 students work on a problem based scenario (e.g. patient with asthma) using role-play with simulated patients in order to practice the Calgary-Cambridge skills related to the scenario. Each group is facilitated by one or two experienced consultation skills tutors.

**Table 1:** Consultation skills programme at the UEA medical school using the Calgary-Cambridge model

MB/BS	Content of Teaching	Length of Time	Student Assessment
**Year 1**	Building the doctor/patient relationship, structuring the consultation, gathering information	~30 hrs of experiential learning 3 hrs lectures	OSCE assessment (formative and summative) Short question and answer assessment of the theory (summative)
**Year 2**	All of the above plus information giving	~15 hrs of experiential learning 3 hrs lectures	OSCE assessment (formative and summative) Short question and answer assessment of the theory (summative)
**Year 3**	All of the above plus shared decision making	~15 hrs of experiential learning 3 hrs lectures	OSCE assessment (formative and summative) Short question and answer assessment of the theory (summative)
**Year 4**	Special circumstances in O&G (Obstetrics & Gynaecology) and paediatrics (e.g. taking a sexual history, breaking bad news, conveying risk)	~27 hrs of experiential learning 7 hrs lectures	OSCE assessment (formative and summative) Short question and answer assessment of the theory (summative)
**Year 5**	Special circumstances in A&E (Accident & Emergency) and Mental Health (e.g. dealing with angry patients, explaining resuscitation orders and advance directives, taking a psychiatric history, assessing mental capacity)	~30 hrs of experiential learning	OSCE assessment (formative and summative)

At the time of the evaluation our tutors comprised the following groups: i) Faculty consultation skills lecturers (two psychologists, one social scientist, one general practitioner, one public health doctor, one ophthalmologist and a senior palliative care nurse). And ii) General practitioners, secondary care clinicians and allied health professionals who work on a sessional basis. All our tutors receive a half-day of training and are paired up with experienced consultation skills tutors until they feel confident and ready to facilitate their own group. They also attend a refresher half-day course as well as other relevant courses every year towards their professional development. Standardisation between groups is encouraged by very detailed teaching plans and tutor handbooks which outline the teaching aims and learning outcomes for every single session.

At the end of the module students' consultation skills are assessed through formative OSCEs and at the end of the year by summative OSCEs [13].

### Evaluation method

A cross-sectional design was used whereby students in all five years of the UEA medical degree (MB/BS) were asked to complete a feedback questionnaire at the end of each experiential consultation skills session they attended during the 2006/7 academic year. There were 639 students studying on the MB/BS course that year. The demographic details of the five year groups at this time can be found in Table 2.

**Table 2:** Demographic characteristics of students enrolled on the UEA MB/BS during the 2006/7 academic year

	Year 1	Year 2	Year 3	Year 4	Year 5
Number of students	167	128	130	110	104
					
Gender:					
*- Male*	57 (34%)	52 (41%)	50 (38%)	45 (41%)	32 (31%)
*- Female*	110 (66%)	76 (59%)	80 (62%)	65 (59%)	72 (69%)
					
Age: Mean (SD)	23.93 (7.18)	22.94 (5.85)	23.82 (6.64)	24.14 (6.88)	24.41 (6.78)
Previous health professional	21 (13%)	25 (20%)	20 (15%)	21 (19%)	16 (15%)
					
Educational background:					
*- Access to Medicine*	33 (20%)	27 (21%)	27 (21%)	20 (18%)	17 (16%)
*- School leaver*	75 (45%)	58 (45%)	62 (48%)	49 (45%)	31 (30%)
*- Graduate*	50 (30%)	43 (34%)	41 (32%)	41 (37%)	56 (54%)

A feedback questionnaire was designed by the consultation skills team to evaluate the consultation skills teaching. The feedback questionnaire is divided into two parts: a quantitative part evaluating the content and process of teaching using 16 closed questions with a 5 point agree-disagree scale, and a qualitative part comprising 5 open-ended questions and space for the students' free comments (Table 3). The evaluation system met Ramsden's criteria [14] regarding characteristics of the evaluating sample, data collection method, and inclusiveness of the evaluation.

**Table 3:** Questionnaire designed to evaluate the consultation skills programme

*Closed questions, scored on a Likert scale from 1 = strongly disagree to 5 = strongly agree*
1) The teaching was clear in its aims and outcomes
2) The content of the tutorials was appropriate for my level of understanding
3) The teaching helped me to make sense of the subject
4) The teaching was relevant to my needs and concerns
5) I had plenty of opportunity to practice consultation skills
6) I was given good feedback on my performance
7) The tutor was happy to answer my questions
8) The tutor's explanations were clear and unambiguous
9) The tutor encouraged my contributions to the tutorial
10) The tutor asked appropriate questions to stimulate thinking
11) The teaching room is well equipped and comfortable
12) The tutor was enthusiastic about working with us
13) The tutor was friendly and approachable
14) The tutor is someone I feel I can trust
15) The tutor was sensitive to my difficulties
16) I would be very happy to work with this tutor again

*Open-ended questions*
17) Were there any difficulties or problems with today's session?
18) What was the most stimulating or challenging part of today's session?
19) What do you feel are the most important things you learnt during this session?
20) What do you feel are the most important things you need to learn more about?
21) Is there any action that you want us to take as a result of today's teaching?

### Data Collection

Ten to fifteen minutes before the end of each experiential consultation skills session the tutor handed out the feedback forms which the students completed anonymously and handed back to the tutor before they left the session. Each tutor collected the forms and handed them to the consultation skills course co-ordinator at the end of the teaching day. Completion of the form was voluntary, but as time was provided during the session most of the students did provide feedback.

Data were collected on 5 occasions in Year 1, 3 in Year 2, 3 in Year 3, 10 in Year 4 and 10 in Year 5. This resulted in 478 evaluation forms from Year 1 students, 359 from Year 2, 326 from Year 3, 926 from Year 4 and 430 from Year 5. Actual attendance figures for each session are not available. So the response rates for year of study refer to the proportion of student session units evaluated, not response rate from attendees (the actual response rate in terms of the number of students who completed the form as a percentage of those who actually attended each session is likely to be higher than reported). Given this, the 2519 forms returned constitute a 67% response rate (57% in Year 1, 93% in Year 2, 84% in Year 3, 83% in Year 4 and 41% in Year 5). Forms were processed and data were entered into Microsoft Excel and exported to SPSS 14.0 for quantitative analysis.

## Results

### Analysis of closed data

Data received in response to the 16 closed questions were subjected to Principal Components Analysis (PCA) using varimax rotation. The aim was to both reduce the number of evaluated characteristics for subsequent analysis, and to investigate which items frequently occurred together. The PCA revealed a three component solution explaining 70% of the variance, with the item referring to the teaching room not loading strongly on any component (Table 4).

**Table 4:** Loadings for the PCA and percentage variance explained for each component

Questionnaire item	Tutor Facilitation (36.86%)	Teaching (20.55%)	Practice & Feedback (12.56%)
The tutor was friendly and approachable	**0.87**	0.19	0.14
I would be very happy to work with this tutor again	**0.84**	0.24	0.14
The tutor is someone I feel I can trust	**0.83**	0.23	0.12
The tutor was enthusiastic about working with us	**0.80**	0.20	0.18
The tutor was sensitive to my difficulties	**0.75**	0.25	0.21
The tutor encouraged my contributions to the tutorial	**0.74**	0.24	0.23
The tutor was happy to answer my questions	**0.73**	0.30	0.23
The tutor's explanations were clear and unambiguous	**0.72**	0.36	0.23
The tutor asked appropriate questions to stimulate thinking	**0.70**	0.33	0.22
The content of the tutorials was appropriate for my level of understanding	0.27	**0.83**	0.15
The teaching helped me to make sense of the subject	0.33	**0.80**	0.21
The teaching was relevant to my needs and concerns	0.26	**0.78**	0.25
The teaching was clear in its aims and outcomes	0.35	**0.76**	0.19
I had plenty of opportunity to practice consultation skills	0.12	0.27	**0.82**
I was given good feedback on my performance	0.21	0.24	**0.81**
The teaching room is well equipped and comfortable	0.19	0.06	0.44

Four subscales were created for subsequent analysis on the basis of those items loading strongly together in the PCA: Tutor Facilitation (the mean of scores for questions 7-10, 12-16), Practice & Feedback (mean for questions 5 & 6), Teaching (mean for questions 1-4) and Teaching Room (mean for question 11).

Analysis using Friedman's ANOVA (assumptions for parametric analysis were not met) indicated that students' evaluation of the different aspects of the consultation skills sessions were significantly different (χ^2 ^(3) = 1263.65, p < 0.001). Post hoc tests using the Wilcoxon signed-rank test (with the significance level corrected to 0.008 using the Bonferroni correction) indicated that students rated the Tutor Facilitation (mean = 4.56, SD = 0.51) most favourably, followed by Teaching (mean = 4.31, SD = 0.59), then Practice & Feedback (mean = 4.14, SD = 0.78), with suitability of the Rooms (mean = 3.72, SD = 1.13) being mostly poorly rated. There were no differences when each of the five year groups of students were considered separately, with the exception that in Year 5 Tutor Facilitation was mostly highly rated, followed by Teaching and Practice & Feedback (no difference between ratings of these aspects of the consultation skills sessions), then Rooms.

### Analysis of student comments

The comments provided by students in response to the open ended question "What do you feel are the most important things you learnt during this session?" were subjected to basic content analysis to summarise and systematise the data. JK summarised the data initially to create a list of phrases or single words that described all the responses given by students. This initial categorisation was followed by merging and refining categories by AP. Finally SM, MF and JK checked the categories for appropriateness and consistency of coding and naming of categories. Disagreements in coding were discussed until all coders agreed.

In answering this question, some students commented on aspects of the consultation skills session that had helped their learning (i.e. **how **they had learnt). Students in all three years noted that the sessions had increased their confidence, particularly through practice. Students in Years 2, 3 and 4 had learnt from reinforcing previous knowledge and skills. Some students in Years 2, 3, 4 and 5 specifically mentioned that they had learnt from "role-play".

Each consultation skills session was designed to cover specific learning outcomes. The skills and knowledge identified by students in response to the question asking them to detail the most important things they had learnt were in line with these specific learning outcomes in many cases. For example, Year 4 students specifically mentioned that they had learnt about how to communicate risk, how to consult with teenagers, and children and their parents; these learning outcomes were specifically covered in Year 4 in line with the teaching they were receiving on paediatrics, obstetrics and gynaecology in the modules for that year. However, there were also marked similarities between multiple years in other areas. For example, all years mentioned that they had learnt about avoiding jargon and how to structure the consultation. Furthermore, there was also some evidence that students in later years felt that they were learning more about aspects of consultation skills that had been covered previously. For example, students in Year 3 identified shared decision making as one of the most important things they had learnt during the session; Year 4 and 5 students had learnt more about this regarding balancing the doctor's and the patient's agenda. This indicates common elements of learning across the five years and spirality of learning (Table 5).

**Table 5:** Skills the students felt they had learnt during the consultation skills sessions

All Years	How to structure the consultation.
All Years	Importance of patient-centredness.

All Years	Aspects of professionalism (including recognising own limits, being prepared, generally acting professionally).

All Years	Avoiding jargon.

All Years	Becoming more reflective.

Year 1	Importance of open and closed questions.

Year 1	Appropriate use of language and non-verbal communication.

Year 1	How to cope with emotions (both patients' and their own), and how to deal with stress.

Year 2, 3 & 4	Checking the patient's understanding.

Year 2, 3 & 4	Giving appropriate information with respect to both amount and type.

Year 2, 3, 4 & 5	Integration of process and content.

Year 2, 3, 4 & 5	Giving and receiving feedback.

Year 3	Shared decision making.

Year 4	How to communicate risk.

Year 4	Not pre-judge patients or use stereotypes.

Year 4	How to consult with teenagers.

Year 4	How to consult with children and their parents. Knowing the right type of question to ask.

Year 4 & 5	Shared decision making - balancing the doctor's and the patient's agenda.

Year 4 & 5	How to deal with three people in a consultation (triadic consultations).

Year 4 & 5	Appropriate use of language and non-verbal behaviour within the context of the specific areas they were covering during their sessions.

Year 4 & 5	How to cope with their patient's emotions and reactions, including breaking bad news.

Year 5	How to consult with patients with mental health problems.

## Discussion

The study shows that students positively evaluated the teaching in all years of the curriculum and were able to identify the skills they thought they had developed, as stated in the curriculum outcomes. These skills increasingly built up on those practiced in earlier years. Tutor facilitation is highly rated by students in all years. This may reflect the care that is given to the selection and training of the facilitators. Consultation skills are integrated into the mainstream curriculum and the scenarios used are congruent with other material that the students are covering at the time. This may contribute to the students' satisfaction with the Teaching.

It is perhaps surprising that the students rate Practice & Feedback less highly than the Tutor facilitation and Teaching. This part of the course involves the active participation of the students and as such is expected to be engaging and effective. Some of the free text comments suggest that this dissatisfaction relates to a desire for more opportunity for practice and feedback. The teaching took place in groups of 10 which limited the opportunity for each individual to be observed interacting with the simulated patient. Further studies are needed to determine the optimal group size and duration of exposure to provide the best learning environment to ensure students get the necessary opportunities to practice and receive individual feedback from the tutor.

The dissatisfaction with the teaching rooms is a local problem which arose because the medical school buildings were incomplete. It is worth noting, however, that the physical environment can have an impact on the students' experiences of learning.

The findings indicate that the learning outcomes were achieved, in that when asked to comment on the most important things they had learnt during the consultation skills sessions, students reported learning skills and knowledge in line with the expected learning outcomes for the session. Although the students were not specifically asked to comment on their perceived self-efficacy (students' confidence in their ability to communicate with patients) and aspects of professionalism (recognising own limits, being prepared, generally acting professionally) they did list these as important outcomes they had learnt. In addition, all years listed structuring the consultation and patient-centredness as amongst the most important aspects of their learning. Relationship building skills such as recognising verbal and non-verbal cues and dealing with emotions were also among the learning outcomes they felt they had achieved from Year 1.

Another important finding is that students in Years 2-5 listed integration of process skills and medical knowledge as a valuable achieved learning outcome which is one of our course's main aims. Core Calgary-Cambridge skills such as giving and receiving feedback when role-playing, balancing the doctor and the patient's agenda, checking the patient's understanding, giving appropriate information with respect to both amount and type and reaching shared decision making were also mentioned as important learning outcomes achieved in the sessions.

The positive learning outcomes achieved by the more advanced students in Years 4 and 5 were also very important. These are the most loaded consultation skills years of the curriculum and require an enormous amount of time and resources. The above findings provide evidence for justifying and maintaining the complexity and intensity of the course in these years. Year 4 and 5 students noted and valued the fact that they had learnt how to communicate risk; to not pre-judge patients or use stereotypes; how to consult with teenagers; how to consult with children and their parents; how to consult with patients with mental health problems; how to cope with triadic consultations; and how to break bad news in different contexts (paediatric, obstetrics and gynaecology, A&E).

The timing of the data collection is both a strength and a weakness of the study. Collecting the feedback at the end of each session enabled students to provide detailed feedback whilst the session was still fresh in their mind; it also enabled collection of feedback about each session individually. In contrast, students might not remember the specifics of each session if the feedback were collected at the end of a rotation or year. Furthermore if feedback was required on each session they had attended in the year they would have to spend considerably more time completing the feedback at that time. An advantage of collecting feedback at the end of the year is that students would have a complete picture of all the teaching they are going to receive that year and would be able to place the individual instances of learning into a wider context.

It is possible that the presence of the Tutor would have influenced the feedback given, with some students feeling that they cannot be as honest as they would like. This was minimised as far as possible by allowing the feedback to be anonymous. Critically, students also complete an annual evaluation of all areas of the MB/BS course in the last third of the year. This evaluation is completed electronically and MB/BS staff receive the feedback anonymously. Whilst this feedback is less specific and does not provide information at an individual session level, it does support the findings of the reported evaluation in terms of the general strengths and weaknesses of the consultation skills teaching identified through the feedback collected at the end of each session.

The cross-sectional nature of the reported study means that we are unable to draw any conclusions directly comparing individual student experience in different year groups as the student proceeds through the course. In the future we hope to investigate how student perceptions of consultation skills training overall from the annual evaluation change over time. Such a longitudinal evaluation of a cohort of students would provide useful information about their experiences of the consultation skills training in the context of what they have learnt in previous years. Whilst questions in evaluation questionnaires are commonly phrased positively, as in the reported study, for the future it would be useful to include some negative statements to see if this has any effect on the student ratings.

There is clearly a need for studies comparing longitudinal and concentrated delivery of consultation skills teaching. In this study, the results suggest that a longitudinal approach to teaching consultation skills allow students to evolve their skills to the point where very sophisticated skills can be learnt. The students develop a clear sense of self-efficacy and feel prepared to deal with difficult communication challenges when they qualify. The current research focuses on subjective reports (student self report) of learning achieved. There is a need to examine whether the student perceptions of what they have learnt match objective assessments of learning. For example, in future work it would be interesting to investigate the relationship between student evaluation of consultation skills sessions and scores on consultation skills stations in the end of module or annual OSCEs (Objective Structured Clinical Examination). This was not possible in the current study as the evaluation data was collected anonymously. Then, the next step would be to find out whether our graduates maintain these complex consultations skills in the clinical environments where they practice and what training they will need in order to continue improving their competence when consulting with patients.

## Conclusions

In summary, consultation skills teaching as presented at UEA using the Calgary-Cambridge model is highly satisfactory to students; it reinforces spiral learning and assimilation of knowledge of the content areas taught. Learning in small groups, the engagement of motivated and trained tutors, and the use of role-play with simulated patients are contributing factors to student satisfaction when teaching consultation skills. The physical environment is a very important factor that could undermine learning if not appropriate to the learning situation and student needs.

## Competing interests

The authors declare that they have no competing interests.

## Authors' contributions

AP contributed to the conception and design of the study. AP, SM, MF and JK contributed to data analysis and interpretation. AP, SM contributed to drafting and critical revision of the manuscript. SJL contributed interpretation of the data and critical revision of the manuscript. All authors approved the final version of the manuscript.

## Authors' Information

Alexia Papageorgiou is a Senior Lecturer and Clinical Communication Theme Lead at St George's, University of London Medical Programme at the University of Nicosia (work conducted whilst at the Norwich Medical School). Susan Miles is a Research Associate in Medical Education, Michelle Fromage is a PhD Student and Sam J Leinster is the inaugural Dean of the Medical School and Professor of Medical Education, all based in the Norwich Medical School at the University of East Anglia. Julie Kemmy is a Senior Advisor at the Norfolk Coalition of Disabled People.

## Pre-publication history

The pre-publication history for this paper can be accessed here:

http://www.biomedcentral.com/1472-6920/11/55/prepub
